# Mast Cells in the Pathogenesis of Multiple Sclerosis and Experimental Autoimmune Encephalomyelitis

**DOI:** 10.3390/ijms131115107

**Published:** 2012-11-16

**Authors:** Massimo Costanza, Mario P. Colombo, Rosetta Pedotti

**Affiliations:** 1 Neuroimmunology and Neuromuscular Disorder Unit, Neurological Institute Foundation IRCCS C. Besta, via Amadeo 42, Milan 20133, Italy; E-Mail: costanza.m@istituto-besta.it; 2 Molecular Immunology Unit, Department of Experimental Oncology and Molecular Medicine, Fondazione IRCCS Istituto Nazionale dei Tumori, via Amadeo 42, Milan 20133, Italy; E-Mail: mario.colombo@istitutotumori.mi.it

**Keywords:** multiple sclerosis, experimental autoimmune encephalomyelitis, mast cells

## Abstract

Mast cells (MCs) are best known as key immune players in immunoglobulin E (IgE)-dependent allergic reactions. In recent years, several lines of evidence have suggested that MCs might play an important role in several pathological conditions, including autoimmune disorders such as multiple sclerosis (MS) and experimental autoimmune encephalomyelitis (EAE), an animal model for MS. Since their first description in MS plaques in the late 1800s, much effort has been put into elucidating the contribution of MCs to the development of central nervous system (CNS) autoimmunity. Mouse models of MC-deficiency have provided a valuable experimental tool for dissecting MC involvement in MS and EAE. However, to date there is still major controversy concerning the function of MCs in these diseases. Indeed, although MCs have been classically proposed as having a detrimental and pro-inflammatory role, recent literature has questioned and resized the contribution of MCs to the pathology of MS and EAE. In this review, we will present the main evidence obtained in MS and EAE on this topic, and discuss the critical and controversial aspects of such evidence.

## 1. Introduction

Multiple sclerosis (MS) is a chronic inflammatory disease of the central nervous system (CNS), characterized by the presence of multifocal plaques of demyelination, immune cell infiltration and axonal damage, primarily located in the white matter [1,2]. It is the most common cause of neurologic disability in the white young adult population, affecting approximately 2.5 million people worldwide [3]. Four clinical patterns of MS have been described [4]. The relapsing-remitting form (RR-MS) affects approximately 85% of patients [4]. It generally starts in the second and third decade of life and has a female prevalence between 2:1 and 3:1, depending on geographical areas [1,5]. RR-MS is characterized by recurrent acute episodes of neurologic disability (relapses) lasting for several days, followed by complete or partial recovery (remissions) over several weeks [1,4]. In approximately 70% of cases, RR-MS converts to a secondary progressive form (SP-MS) in later stages of disease [1]. Early symptoms of RR-MS include unilateral optic neuritis, double vision (diplopia), sensory disturbances, limb weakness, ataxia [1,3]. In more advanced stages of disease cognitive deficits (e.g., memory loss, impaired attention), dysphagia, progressive quadriparesis and sexual dysfunction can occur. Cortical signs (early dementia, aphasia, seizures) are occasionally present in MS [1,3]. In 10% of patients the disease is progressive from the onset without relapses, therefore called primary-progressive (PP-MS), and displays a similar incidence between females and males [4]. Approximately 5% of patients suffer from a progressive-relapsing form of disease (PR-MS), characterized by a progressive onset, associated to one or more relapses in later stages of disease [4].

MS is widely thought to occur in genetically predisposed individuals after exposure to an environmental trigger that activates myelin-specific T cells in peripheral lymphoid organs. Following re-stimulation in the CNS, autoreactive T cells orchestrate an immune-mediated attack against components of myelin, inducing demyelination and axonal injury [6]. Elements of both acquired and innate immune responses are involved in this process. Demyelination and axonal injury lead to inefficient propagation of action potentials through the internodes of nerves (loss of saltatory conduction) and result in neurological deficits [3]. MS and EAE, the animal model for this disease, are generally perceived as CD4^+^ T helper 1 (Th1)/Th17-mediated autoimmune diseases [7]. However, several lines of evidence in recent years suggest that immune components and mechanisms associated with Th2-driven “allergic” disorders may take part to the development of CNS autoimmunity [8,9]. Among those, mast cells (MCs), which represent the key effectors cells in IgE-mediated immediate hypersensitivity reactions, have also been implicated in the development of MS and EAE [10]. Since their first description in MS plaques in 1890 by Neuman [11] and almost a century later by Olsson [12], a large body of literature has explored the involvement of MCs in the pathogenesis of MS and EAE. In both humans and rodents, the localization of MCs in the leptomeninges has initially prompted to speculate a possible contribution of these cells in regulating the trafficking of immune cells through the blood-brain barrier (BBB) [13,14]. Indeed, meningeal vasculature represents one of the first sites of arrest of autoreactive T cells infiltrating the CNS [15]. Further studies have implicated also an immunomodulatory role of MCs occurring in peripheral lymphoid organs [16,17]. However, today the exact role of MCs in CNS autoimmune disease is highly debated, in particular with regard to data obtained in animal models, which have often shown contradictory outcomes between different groups. In this review, we will provide a general overview on MC biology before focusing on the main pieces of evidence involving MCs in the pathology of MS and EAE and highlighting discrepancies and critical data available on this topic.

## 2. Biology of Mast Cells

### 2.1. Development and Phenotypes

MCs are components of the innate immune system arising from multi-potent hematopoietic progenitors cells, and are phenotypically identified for high expression on their surface of the tyrosine kinase receptor c-*kit* (CD117) and the high-affinity Fc receptor for IgE (FcɛRI) [18]. In mice, they have been proposed to derive from a specific MC progenitor, distinct from common myeloid progenitors or granulocyte/macrophage progenitors of the adult haematopoietic pathway [19]. MCs circulate in the blood as precursor cells and undergo maturation in peripheral tissues. Unlike basophils, MCs are long-lived (weeks to months) and exhibit a certain degree of proliferative potential also following differentiation [20]. They reside in most tissues, strategically located in proximity of epithelial barriers exposed to environmental triggers, such as the skin, airways and gastrointestinal tract. This location sets MCs in a particularly relevant position for the initiation and propagation of immune responses [21]. This property is well exemplified in mouse models of cutaneous contact hypersensitivity reaction, where MCs have been proved to promote dendritic cells migration from the skin to the draining lymph node (DLN) and sustain hypercellularity of DLN [22]. A novel proposed mechanism through which activated MCs signal from peripheral inflamed tissue to lymphoid organs is the secretion of insoluble heparin-based particles containing tumor necrosis factor (TNF) and proteases, which are drained through lymphatics to the lymph node and promote hypertrophy of the lymphoid tissue [23]. MCs have been also shown to migrate to DLN through C-X-C chemokine receptor type 4 (CXCR4), supporting systemic immune suppression induced by ultraviolet irradiation of the skin [24].

Stem cell factor (SCF), also known as the ligand for c-*kit*, is the main growth factor for MC development, although interleukin (IL)-3, IL-4, IL-9 and transforming growth factor (TGF)-β can also modulate the number, phenotype and function of MCs [25,26].

Based on the anatomical distribution and/or granule content, rodent MCs have been classified in two different subpopulations: mucosal MCs (MMCs), residing in the respiratory and gastrointestinal tracts, and connective tissue-type MCs (CTMCs), located in the skin, peritoneal cavity and connective tissue. MMCs are generally smaller than CTMCs and they differ from each other for the content of proteases, proteoglycans and histamine within their granules [18,25]. In humans, the presence of tryptase or both tryptase and chymase in MC granules is used to distinguish two subsets, tryptase MC (MC_T_), identified in the lung and intestinal mucosa, and tryptase/chymase MC (MC_TC_), which is found in the skin [18,25]. The phenotype of MCs seems to be plastic and rely on the specific microenvironment of their tissue of residence. In rodents, it has been demonstrated that a peritoneal CTMC transplanted into the stomach wall of a MC-deficient mouse can acquire the histologic and electron microscopic traits of a MMC after seeding in the mucosa, while retaining the features of a CTMC in the muscularis propria of the same organ [27].

### 2.2. Activation and Immune-Modulating Functions

MCs express a wide array of receptors, which allow them to “sense” the microenvironment and finely respond to different kind of stimuli. The best characterized mode of MC activation is the IgE-mediated immune reaction. The cross-linking of FcɛRI-bound IgE with a multivalent antigen induces aggregation of two or more FcɛRI molecules and activates downstream intracellular-signaling events leading to degranulation and synthesis of new mediators [28]. MC-granules contain biogenic amines (histamine and, only in rodents, serotonin), serglycin proteoglycans (heparin and chondroitin sulphate), serine proteases (tryptases, chymases and carboxypeptidases), cytokines (such as TNF-α) and growth factors (such as vascular endothelial growth factor A (VEGFA)) [29]. FcɛRI-mediated activation of MCs induces also the *ex novo* synthesis of lipid mediators as prostaglandins (PGD_2_, PGE_2_) and leukotrienes (LTB_4_, LTC_4_), cytokines (e.g., TGF-β, IL-4, IL-10), chemokines (such as CC-chemokine-ligand 2) and growth factors (e.g., nerve growth factor (NGF) [26,30]. IgE alone can increase MC survival and promote the production of cytokines such as IL-4, IL-6 and TNF-α [31]. Furthermore, in mice MCs can be induced to degranulate by antigen-IgG_1_ through FcγRIII [32,33]. Myelin proteins such as myelin basic protein (MBP) can activate rat MCs [34,35] through interaction with scavenger receptors [35]. MC activation by MBP has also been shown to induce neurotoxicity in mixed hippocampal cultures [36]. This neurotoxic effect of MCs was reduced by treatment with palmitoylethanolamide, an endogenous anti-inflammatory fatty acid amide involved in autacoid local injury antagonism (ALIA) [36,37]. MCs express numerous receptors for other ligands—such as cytokines, chemokines, complement component 3a (C3a), C5a and pathogen-associated molecular patterns (PAMPs)—which can either support FcɛRI-mediated MC activation or foster the secretion of selective mediators [28]. For example, lipopolysaccharide (LPS) activation of toll-like receptor (TLR)-4 stimulates the release of IL-6 rather than preformed granule-associated mediators [38]. Activation of TLR-2 results in preferential secretion of pro-inflammatory cytokines (e.g., IL-6, IL-17 and interferon (IFN)-γ) [39]. The nerve growth factor, which is stored and released also by MCs [40], can modulate MC function [41]. Of interest in the context of MS, NGF has been found increased in the CSF of patients during acute attacks of disease [37,42] and both MCs and NGF have been reported to increase in chronic inflammatory states such as MS [37].

Depending on the encounter with specific inflammatory milieu and selective stimuli, MCs have been shown both *in vivo* and *in vitro* to exert different or even opposite functions in biological responses. This peculiarity is exemplified by the interaction between MCs and Foxp3^+^ regulatory T cells (Treg), a T cell subset essential in the maintenance of immune tolerance and limitation of autoimmunity [43]. In an experimental model of tolerant skin allograft in mice, MCs have been reported to support allograft acceptance by establishing a bi-directional, functional cross-talk with Treg, which recruited and activated MCs in tolerant tissue through secretion of IL-9 [44]. However in the same model, if MCs were induced to degranulate by IgE-Ag or chemically by compound 40/80, they promoted rejection of the established tolerant allograft, and transient impairment of Treg suppressive function [45]. Notably, other inflammatory stimuli, such as LPS, Complete Freund’s Adjuvant (CFA)—an adjuvant consisting of killed *Mycobacterium tuberculosis* in paraffin oil, commonly used to elicit EAE—and CpG-ODN (a TLR-9 agonist) were not able to trigger acute rejection of skin transplant [45].

MCs stimulated *in vitro* with LPS or IFN-γ have been shown to process and present Ag to T cells, with preferential expansion of Ag-specific Treg over naïve T cells, suggesting that under these specific conditions of activation MCs might be supportive for Treg populations [46]. Conversely, in a different *in vitro* setting, others and we have shown that MCs unstimulated or activated with IgE or IgE-Ag were able to break Treg suppressive capacity by IL-6 and OX40-dependent mechanisms [47] or through secretion of histamine [48]. Also, as part of the reciprocal cross-talk, Treg can suppress FcɛRI-dependent MC degranulation through the OX40-OX40L interaction [49].

Because of the plasticity and complexity of MCs responses, they have been suggested to play roles in autoimmune diseases, including MS and its animal model EAE. In these disorders, depending on the specific pathological context under investigation, MC has been proposed to either enhance or dampen self-reactive immune responses. In an experimental model of bullous pemphigoid, an autoantibody-associated disorder of the skin, MCs promoted neutrophil infiltration and subepidermal blistering in the inflamed tissue [50]. Conversely, in mouse models of immune complex-mediated nephritis, MCs have been suggested to protect from diffuse proliferative glomerulonephritis [51] and to increase survival by limiting glomerular injury, reducing T cells and macrophage infiltration at inflammatory sites, and by promoting remodeling of renal tissue [52,53]. MCs have also been proposed as detrimental immune players in the pathogenesis of autoimmune arthritis [54,55]. However, their exact contribution to this disease has been recently challenged by several studies [56,57] (see Section 3.2.3 for detailed discussion).

## 3. Mast Cells in CNS Autoimmunity

### 3.1. Mast Cells in Multiple Sclerosis

After the first description of MCs in MS plaques in 1890 [11], several neuropathological studies have subsequently confirmed and detailed their presence in MS brain [12,14,58–61]. MCs have been detected within demyelinated lesions, often in perivascular areas associated with immune cell infiltrates, but also in the CNS parenchyma [14,58,59,61]. Remarkably, MCs resembled the CTMC phenotype and did not show any sign of degranulation [14]. Also, they were more frequently observed in chronic-active plaques than in acute lesions [14]. In line with these findings, gene microarray and real time PCR analyses of chronic MS lesions revealed an up-regulation of MC-associated genes such as tryptase, chymase and FcɛRI β chain [61,62]. Interestingly, one report found that transcripts of tryptase and chymase were overexpressed also in the normal appearing white matter of MS patients [61]. In addition, the concentration of MC tryptase was found significantly higher also in the cerebrospinal fluid of MS subjects [63].

Collectively, these findings suggested that MCs might play a role in the pathogenesis of MS, and prompted several studies in experimental models aimed at elucidating their involvement in CNS autoimmune disease.

### 3.2. Mast Cells in Experimental Autoimmune Encephalomyelitis

The potential mast cell contributions to human MS have been widely investigated by taking advantage of EAE, an extensively used animal model for this disease [64]. EAE can be elicited in a wide range of species, but most commonly in rats and mice [65]. It is elicited in susceptible strains by active immunization with immunodominant epitopes of myelin antigens supplemented with adjuvants such as CFA and *Bordetella pertussis* Toxin (PTX) [66,67]. In the majority of the models, EAE clinically manifests as an ascending-flaccid paralysis starting in the tail and progressing to the hind and forelimbs [67]. In C57BL/6 mice (bearing H-2^b^ haplotype of major histocompatibility complex (MHC)), EAE can be induced by subcutaneous administration of myelin oligodendrocyte glycoprotein peptide 35–55 (MOG_35–55_) in CFA and by intravenous or intraperitoneal injection of PTX. These mice develop EAE with a chronic clinical course of paralysis [68]. Immunization with proteolipid protein peptide 139–151 (PLP_139–151_) of SJL-J mice (H-2^s^) results in a relapsing-remitting form of EAE [69].

Active EAE comprises an induction phase, which involves the priming and activation of myelin-specific CD4^+^ Th1/Th17 cells in peripheral lymphoid organs, and an effector phase, during which encephalitogenic CD4^+^ T cells migrate into the CNS, are re-activated by APCs and orchestrate an immune-mediated attack against myelin. EAE lesions are infiltrated by macrophages, CD8^+^ T cells, B cells and plasma cells, resembling the neuroinflammatory milieu observed in MS plaques [70]. The effector phase of disease can also be studied by passive EAE, which is obtained by transfer of previously activated myelin-specific T cells into recipient animals [71].

MCs have been hypothesized to take part to both induction and effector phases of EAE, by modulating the autoimmune response in peripheral lymphoid organs and/or regulating the access of immune cells into the CNS.

#### 3.2.1. Histopathological Characterization of Mast Cells in EAE

Several histopathological studies have examined the frequency and distribution of MCs in both CNS and peripheral lymphoid organs during the course of EAE in different animal species. Some work reported a decrease in the number of MCs in dura mater [72], velum interposition [72] and thalamus [34,73] during the acute phase of EAE in Lewis rats, while other described no change [74] or a three-fold rise in thalamic MCs [75]. Conversely, most of the studies were concordant in describing an increase of the percentage of degranulated MCs in the brain of EAE rats [34,74,75], thus proposing that MCs might be involved in the effector phase of the disease. In the CNS of naïve mice, MCs have been identified in perivascular areas of leptomeninges, hippocampus, habenula and thalamus [76,77]. During the course of mouse EAE, no degranulated MCs or MCs infiltrating acute lesions were detected in either WBB6F_1_ or C57BL/6 strains [77,78]. In marmoset EAE, MC activation was increased in areas of demyelination in the diencephalon [79]. Indeed, in this model MCs were located in perivascular areas and displayed ultrastructural evidence of intragranular activation (indicating the release of selective mediators), but not degranulation [79].

The histological evaluation of peripheral lymphoid organs during the induction phase of EAE in C57BL/6 mice, revealed a greater number of MCs within the T-cell-rich perifollicular areas of DLN, with a certain degree of MC-clustering [78], and the presence of activated MCs establishing tight spatial interactions with Th17 cells and regulatory T cells [47]. These findings again evoked the occurrence of a potential MC-mediated modulation of Treg and Th17 cells immune functions [47].

#### 3.2.2. Pharmacological Targeting of Mast Cells in EAE

The first studies attempting to clarify the role of MCs in EAE sought to modulate disease development by treatment with pharmacological agents able to block or induce MC degranulation. In 1989, Dietsch *et al.*, showed that the incidence of passive EAE in Lewis rats was drastically abated if animals were treated intraperitoneally just before disease onset with proxicromil, a MC-stabilizer derivative of cromolyn [80]. They also reported that reserpine, a pharmacological agent inhibiting MC release of vasoactive amines, was effective in reducing the incidence of both active and passive EAE in Lewis rats [80]. However, a few years later, Levi-Schaffer and co-workers demonstrated that another derivative of cromolyn, nedocromil, was just efficacious in slightly delaying EAE onset in rats, and if administered at the time of disease induction, thus suggesting that MCs were only partially involved in the priming phase of disease, and perhaps dispensable for the effector phase [73]. Nevertheless, another group showed that intracisternal but not intraperitoneal administration of compound 48/80, which triggers MC degranulation, also reduced EAE severity in rats, underscoring the possibility of MC contribution to disease development in the CNS [72].

Since these first initial works, the involvement of MCs in EAE appeared somehow ambiguous. Considering that all these pharmacological agents are not MC specific being active also on other cell types [81,82], no direct conclusions could be drawn on the role of MCs in EAE by these pharmacological studies.

#### 3.2.3. EAE in Mast Cell-Deficient Mouse Models

In recent years, a significant amount of work has attempted to assess MC involvement in EAE by using mouse strains harbouring spontaneous inactivating mutations of c-*kit* gene (or, in C57BL/6-*Kit*^W-sh/W-sh^ mice, a mutation that reduces c-*kit* expression [see below]) and consequently displaying severe MC deficiency [83,84]. The availability of c-*kit* mutant MC-deficient mouse models has provided the opportunity of applying an apparently more specific experimental approach to study MCs in EAE. However, data obtained with these mice appear often discordant and/or contradictory, and have depicted an equivocal and conflicting scenario about the exact impact of MCs in CNS autoimmunity.

For several years the most commonly used model for studying MCs has been the *Kit*^W/W-v^ strain on WBB6F_1_ background [25,26]. WBB6F_1_-*Kit*^W/W-v^ mice bear two mutated alleles at the White spotting *(W)* locus on chromosome 5, which corresponds to c-*kit* gene. The W mutation is a G to A point mutation at a splice donor site leading to exon skipping and production of a truncated c-*kit*, which lacks the transmembrane domain and is not expressed on the cell membrane [85]. The W-v mutation is a C to T point mutation (resulting in the change Thr660Met) in the c-*kit* tyrosine kinase domain that considerably reduces receptor signalling [86,87]. *Kit*^W/W-v^ mice display profound MC-deficiency, but also some other c-*kit*-dependent abnormalities, such as defective melanogenesis, sterility, anemia, deficiency of interstitial cells of Cajal (ICCs) and neutropenia [88].

The group of M. Brown, first in 2000, reported that MC-deficient WBB6F_1_-*Kit*^W/W-v^ mice developed MOG_35–55_-induced chronic EAE with a significantly lower incidence and milder severity than controls. Engraftment of *Kit*^W/W-v^ mice with bone marrow-derived, *in vitro* cultured MCs (BMMCs) before EAE induction, restored disease susceptibility to levels of wild-type mice, thus clearly indicating a detrimental role of MCs in this model [77]. Activation of BMMCs through the Fc receptor common γ-chain (shared by FcγRI, FcγRIII and FcγRI) or through FcɛRIII was essential to promote EAE in this model [89]. Further studies by the same group have outlined that MCs influenced disease development by acting both in peripheral lymphoid organs [16] and the CNS [90]. Indeed, MCs were proposed to be necessary for the establishment of an optimal encephalitogenic Th1 cell response in both lymph nodes and spleen [17]. Adoptive transfer of myelin-activated T cells also resulted in less severe EAE in *Kit*^W/W-v^ mice compared to controls [17]. In the CNS, meningeal MCs were suggested to contribute to the breach of the BBB occurring in EAE, by favouring the recruitment of neutrophils into the CNS parenchyma through secretion of TNF [90]. Recently, Brown and co-workers have shown that SJL/J-*Kit*^W/W-v^ mice displayed attenuated PLP_139–151_-induced EAE, thus indicating that MCs may promote also the relapsing-remitting model of MS [91].

Although these results obtained in mouse models of MC deficiency have highlighted an important contribution of MCs to the development of EAE, other studies have recently questioned these data, showing that EAE in WBB6F_1_-*Kit*^W/W-v^ developed with no significant difference or even with higher severity compared with wild-type littermates [57,78,92]. The reasons for these discrepant results are still to be understood. However, in the original work by Secor *et al.*, showing that *Kit*^W/W-v^ mice were protected from EAE [77], the protocol of disease induction was much stronger than those generally used to induce EAE, and consisted of 300 μg of MOG_35–55_ emulsified in 500 μg of *M. tuberculosis* in CFA (injected on days 0 and day 7 post-immunization) and 500 ng of PTX (on days 0 and 2 p.i.). In the two papers reporting full susceptibility of *Kit*^W/W-v^ mice to EAE, the disease was elicited by administration of lower amounts of peptide and adjuvants (*i.e.*, 200 μg of MOG_35–55_ in 550 or 800 μg of *M. tuberculosis* in CFA (on day 0) and 200 ng of PTX (on days 0 and 2 p.i.) [57,92]. We have tried somehow to reconcile the divergent EAE outcomes obtained in different works by proposing that EAE expression in *Kit*^W/W-v^ model was “tunable” according to the doses of peptide and adjuvants used to elicit EAE. Indeed, we have demonstrated that *Kit*^W/W-v^ mice developed milder EAE than controls only when immunized with a “strong” protocol of immunization (*i.e.*, similar to the one used by Secor *et al*. [77]) [78]. Conversely, when a low/normal protocol of immunization was used (*i.e.*, 100 μg of MOG_35–55_ in 200 μg of CFA on day 0 and 200 ng of PTX on days 0 and 2 p.i.) EAE was actually slightly exacerbated in *Kit*^W/W-v^ mice [78]. Indeed, the reliance on the specific experimental setting observed in this strain is common to animal models of asthma, contact hypersensitivity and bacterial infection, where the induction protocol can drastically affect the importance of MC’s contributions to the disease model under investigation [93–95]. It can be hypothesised that diverse experimental conditions/protocols for disease elicitation may result in different pathological mechanisms, which might impact on the same mutation in alternative ways. However, a more recent report from Brown’s group has described reduced EAE severity in *Kit*^W/W-v^ mice even upon the application of a relatively mild immunization protocol (100 μg of MOG_35–55_ in 5 mg/mL CFA and 250 ng of PTX) [90], rendering the interpretation of such discrepancies unclear. Based on the results of Brown *et al.*, it seems that different protocols of EAE induction appear not to be the only factor involved in the divergent results obtained by different groups with *Kit*^W/W-v^ mice.

This controversial picture has been further complicated by data produced on a more recently tested c-*kit* mutant MC-deficient strain, the C57BL/6-*Kit*^W-sh/W-sh^ mouse. The W-sash (W-sh) mutation consists of an inversion mutation upstream from the c-*kit* promoter, covering approximately 3Mb and including 27 genes. The 3′ end of this inversion breaks a regulatory locus that controls c-*kit* expression specifically in MCs, whereas the 5′ breakpoint is localized between exons 5 and 6 of *corin* gene, which as a result is disrupted [96,97]. *Kit*^W-sh/W-sh^ mice exhibit severe MC-deficiency, lack melanocytes and ICCs, but they are not anaemic nor sterile, unlike the *Kit*^W/W-v^ animals [88]. Nevertheless, they are affected by some other hematopoietic alterations such as splenomegaly with expanded myeloid populations, and an increased number of circulating neutrophils, platelets [97] and basophils [94].

Although in a first report *Kit*^W-sh/W-sh^ mice were described to develop milder EAE compared to control mice [98], we and others have independently shown in subsequent work that, surprisingly, EAE in C57BL/6-*Kit*^W-sh/W-sh^ mice was exacerbated compared to that in control mice with MCs, with an earlier disease onset and a more severe progression compared to sibling controls [78,99]. *Kit*^W-sh/W-sh^ mice also displayed more severe EAE under different conditions of immunization [78]. Bennett *et al*. reported no significant clinical difference in EAE between *Kit*^W-sh/W-sh^ and *Kit*^+/+^ mice [92]. Nevertheless, all of these studies were concordant in describing a more pro-inflammatory profile of autoreactive T cells in peripheral lymphoid organs of *Kit*^W-sh/W-sh^ animals. Indeed, myelin-specific T cells from MC-deficient mice exhibited an increased proliferative response to MOG_35–55_[78,92,99], enhanced secretion of Th1/17 cytokines such as IFN-γ, IL-6 and IL-17A, and a decreased production of Th2 or suppressor cytokines, such as IL-4, IL-5 and IL-10 [78,99]. Higher clinical severity was also associated with a reduction of Treg frequencies in the spleen [78] or the CNS [99] of *Kit*^W-sh/W-sh^ mice.

Mast cell knock-in studies have also been conducted to verify the contribution of MC to the EAE output observed in *Kit*^W-sh/W-sh^ mice. In our work, intravenous transplantation of BMMCs in *Kit*^W-sh/W-sh^ mice 6–8 weeks before EAE induction (in line with common procedures for performing MC-knock-in experiments [20,100]) was not effective in restoring EAE severity to wild-type mice levels [78]. In this setting of engraftment, MCs engrafted only partially the priming sites, (*i.e.*, the inguinal and axillary lymph nodes) but not the CNS, as also observed in previous work [88,100]. Nonetheless, in these conditions we could verify the rescue of some MC-related biological functions, such as normal percentages of Treg and granulocytes in lymph nodes and spleens, respectively, of the MC-engrafted mice. However, this engraftment setting did not allow us to evaluate the contribution of MCs to EAE development into the CNS. By using an “alternative” MC-engraftment experiment, Li *et al.* showed reversion of increased EAE severity of *Kit*^W-sh/W-sh^ mice [99]. Indeed, they injected BMMCs into *Kit*^W-sh/W-sh^ animals just before EAE onset. BMMCs-transplanted *Kit*^W-sh/W-sh^ exhibited EAE severity, frequency of Treg in the CNS and peripheral myelin-specific immune response comparable to those observed in wild-type mice. Remarkably, in this MC-knock-in setting, MCs were found also in the CNS [99]. It is possible that the enhanced immune cell-infiltration in the CNS of *Kit*^W-sh/W-sh^ mice [78,99] might have been the effect of an exacerbated peripheral activation of immune cells but also of their increased trafficking (and/or re-activation) through the BBB into the CNS occurring in absence of MCs. Of note, passive transfer of myelin-activated T cells resulted in earlier EAE onset in *Kit*^W-sh/W-sh^ mice compared to controls [78], again suggesting a possible impact of *Kit*^W-sh/W-sh^ mutation and/or MCs directly in the effector phase of the disease occurring in the CNS.

Taken together, the data obtained in the *Kit*^W-sh/W-sh^ model indicated that MCs might be dispensable for, or even limit, the establishment of anti-myelin T cell responses in both peripheral lymphoid organs and the CNS, regardless the conditions of immunization. Interestingly, we have shown that histidine decarboxylase (HDC)^−/−^ mice, carrying histamine deficiency but also MC paucity, develop EAE with earlier onset and extensive granulocytic infiltration of the CNS [101]. This phenotype resembles somehow the clinical and histological outcome of MC-deficient *Kit*^W-sh/W-sh^ mice, which bear just about one-third of wild-type histamine levels in the brain [102]. In this regard, it should be considered that in the context of CNS autoimmunity, histamine has been demonstrated to reduce BBB permeability by stimulating histamine receptor 3 (H3R) on brain presynaptic neurons and histamine receptor 1 (H1R) on brain endothelial cells [103,104]. In addition, histamine has been shown to reduce the firm arrest of encephalitogenic T cells to the inflamed brain circulation in an *in vivo* model of early EAE inflammation [105]. Thus, it could be speculated that reduced levels of histamine of *Kit*^W-sh/W-sh^ mice might contribute, in part, to EAE phenotype and autoreactive T cell responses observed in these mice.

Collectively, the works discussed above depict an ambiguous scenario about the involvement of MCs in the pathogenesis of EAE. Indeed, in c*-kit* mutant models, a certain variability in disease outcome in the same MC-deficient strain as well as in *Kit*^W/W-v^*versus Kit*^W-sh/W-sh^ mice has been observed. On the whole, the expression of EAE in the *Kit*^W/W-v^ model appears to a certain extent to be affected by the immunization conditions, while in *Kit*^W-sh/W-sh^ mice, developing similar or more severe EAE compared to wild type mice, there was a trend toward an exacerbated anti-myelin pro-inflammatory T cell response. Different results obtained in the same MC-deficient model may reflect MCs plasticity and their “tunable” response to different kind or amount of stimuli, but may also depend on mouse housing conditions, gut micro flora composition or other reasons that still need to be elucidated. Divergences between *Kit*^W/W-v^ and *Kit*^W-sh/W-sh^ models may depend on genetic background or may be the result of different and complex hematopoietic alterations of these mice. Indeed, MC-engraftment via intravenous route has been shown not to recapitulate in MC-deficient mice the distribution and amount of MCs observed in wild-type mice [20]. Even though in some cases MC-engraftment was sufficient to recover some biological responses to wild-type levels [78], it cannot be ruled out that MCs may play an aberrant and not-physiologic role in the context of severe immune alterations, such as the neutropenic or neutrophilic status of *Kit*^W/W-v^ and *Kit*^W-sh/W-sh^ mice, respectively. In this regard, studies on models of antibody-mediated arthritis have provided a straight example of how granulocytes abnormalities of *Kit* mutant strains might impact the development of immune responses. Initially *Kit*^W/W-v^ mice were shown to be resistant to arthritis induced by injection of antibodies (Abs) to glucose 6-phosphate isomerase and MC-secreted IL-1β was proposed to promote joint inflammation in this model [54,55]. A subsequent study demonstrated that, surprisingly, *Kit*^W-sh/W-sh^ but not *Kit*^W/W-v^ mice were fully susceptible to arthritis induced by Abs to type II collagen and LPS [56]. Also, by depleting Gr1^+^ immune cells, authors had shown that granulocytes, rather than MCs, were playing a major role of in the pathogenic mechanisms driving tissue damage in this model, suggesting that neutropenic status of *Kit*^W/W-v^ mice was actually responsible for their resistance to disease [56].

A valuable effort to elucidate MC contribution to EAE pathogenesis has been recently made by Feyerabend *et al.*[57], who evaluated EAE in a novel mouse model of MC-deficiency, independent of c-*kit* abnormalities. In this strain, the insertion of a Cre-recombinase into the mast cell carboxypeptidase A3 (Cpa3) locus by targeted recombination resulted in selective deletion of MC-lineage, due to the genotoxic effect of sustained Cre expression [57]. Analysis of MC frequency in heterozygous *Cpa3^Cre/+^* mice (on a C57BL/6 background) revealed ablation of both mucosal and connective-tissue MC populations in the intestine, skin and peritoneal cavity, and a partial reduction of splenic basophils [57]. *Cpa3^Cre/+^* mice developed EAE with clinical severity, CNS infiltration by immune cells and peripheral anti-MOG_35–55_ T cell response comparable to wild-type mice. Of note, *Kit*^W/W-v^ mice developed EAE with the same clinical severity of *Cpa3*^Cre/+^ and *Cpa3*^+/+^ mice, even though they were not compared to *Kit*^+/+^ control littermates [57]. Collectively, data obtained in MC-deficient *Cpa3*^Cre/+^ model have indicated that MCs are neither promoting nor dampening CNS autoimmune response occurring in EAE and play a redundant role in the clinical expression of disease. Interestingly, this study also demonstrated that *Cpa3*^Cre/+^ mice developed full antibody-mediated arthritis with no significant difference compared to *Cpa3*^+/+^ mice, suggesting that MCs are dispensable for development of arthritis and that c-*kit* mutation rather MC deficiency was responsible for disease resistance in *Kit*^W/W-v^ strain [57].

## 4. Conclusions

Broad evidence obtained through MS and EAE has suggested that MCs might be involved in CNS autoimmunity. In MS and EAE, MCs have been hypothesized to exert their functions within the CNS, where they might modulate trafficking of inflammatory cell through the BBB, and/or in peripheral lymphoid organs, where they could modulate autoreactive T cell responses. However, studies on EAE performed with c*-kit* mutant strains have produced conflicting results. In the *Kit*^W/W-v^ mouse model, MCs have been shown to promote EAE pathology only in specific experimental settings (*i.e.*, high doses of peptide/adjuvant in immunization protocol) [77,78], while in other experimental conditions MCs were shown to play a redundant role, or even to reduce disease severity [57,78,92]. Conversely, most of the work performed in the *Kit*^W-sh/W-sh^ mouse model pointed out a potential role of MCs in limiting anti-myelin pro-inflammatory T cell responses [78,92,99] and disease severity [78,99]. The reasons for these discrepancies still need to be understood. Phenotypic abnormalities in these mice other than their MC-deficiency may also have contributed to EAE phenotype observed in these models. The new *Cpa3*^Cre/+^ strain represents a novel model for evaluating MC function in certain diseases and might represent a valuable tool to address the involvement of MC in EAE in absence of c-*kit*-dependent phenotypic abnormalities. The data produced in this mouse model have so far suggested that MCs do not contribute to EAE. However, it should be remembered that these mice display a reduction in splenic basophils, whose potentially important role in autoimmunity has recently been identified [106]. Moreover, the absence of MCs in *Cpa3*^Cre/+^ mouse model should be ascertained also in the CNS, before concluding that MCs are dispensable for EAE. Furthermore, given the complexity and variability of the results produced in *c-kit* mutant mice, it may be interesting to explore in the new *Cpa3*^Cre/+^ mouse model of MC-deficiency the effects of differential dosing of the immunization protocol on the clinical course of EAE.

## Figures and Tables

**Table I:** Experimental autoimmune encephalomyelitis (EAE) outcomes in mast cell (MC)-deficient strains under different conditions of immunization.

Strain	Immunization protocol	EAE severity	References
*WBB6F_1_-Kit*^W/W-v^	300 μg of MOG_35–55_ plus 500 μg of *M.T.* (days 0, +7)	Reduced	[16,17,77,78,89]
	200 μg of MOG_35–55_ plus 800 μg of *M.T.* (day 0)	No difference	[92]
	100 μg of MOG_35–55_ plus 5 mg/mL of *M.T.* (day 0)^1^	Reduced	[90]
	100 μg of MOG_35–55_ plus 200 μg of *M.T.* (day 0)	Worsened	[78]
	200 μg of MOG_35–55_ plus 550 μg of *M.T.* (day 0)	Susceptible 2	[57]

*C57BL/6-Kit*^W-sh/W-sh^	200 μg of MOG_35–55_ plus 800 μg of *M.T.* (day 0)	No difference	[92]
	200 μg of MOG_35–55_ plus 4 mg/mL of *M.T.* (day 0)^1^	Reduced	[98]
	200 μg of MOG_35–55_ plus 400 μg of *M.T.* (day 0)	Worsened	[78]
	300 μg of MOG_35–55_ plus 500 μg of *M.T.* (days 0, +7)	Worsened	[78]
	200 μg of MOG_35–55_ plus 4 mg/mL of *M.T.* (day 0) 1	Worsened	[99]

*SJL-Kit*^W/W-v^	100 μg of PLP_139–151_ plus 5 mg/mL of *M.T.* (day 0) 1	Reduced	[91]

*C57BL/6-Cpa3*^Cre/+^	200 μg of MOG_35–55_ plus 550 μg of *M.T.* (day 0)	No difference	[57]

Abbreviations: M.T., Mycobacterium Tuberculosis;

1 Final volume of the emulsion not specified;

2 In this paper *Kit*^W/W-v^ mice are shown to develop severe EAE and are compared to Cpa3^+/+^ and Cpa3^+/−^ but not to WBB6F_1_-*Kit*^+/+^ mice.
